# Survival machine learning models for predicting all-cause and case-specific mortality risk in metabolic dysfunction-associated fatty liver disease patients

**DOI:** 10.1038/s41598-025-26729-z

**Published:** 2025-11-28

**Authors:** Jingpeng Gao, Nan Zhang, Akemujiang Aximu, Ning Xin, Ziwei Wang, Ping Yan

**Affiliations:** 1 Department of Infectious Disease, General Hospital of Xinjiang Military Command, Urumqi, Xinjiang China; 2 State Key Laboratory of Pathogenesis, Prevention and Treatment of High Incidence Diseases in Central Asia, Xinjiang Medical University, Urumqi, Xinjiang China; 3 School of Nursing, Xinjiang Medical University, Urumqi, Xinjiang China; 4 Xiangya School of Nursing, Central South University, Changsha, Hunan China; 5 Health Care Research Center for Xinjiang Regional Population, Urumqi, Xinjiang China; 6 Department of Thoracic Surgery, PLA 960th Hospital, Jinan, Shandong China; 7 Emergency Center, People’s Hospital of Xinjiang Uygur Autonomous Region, Urumqi, Xinjiang China

**Keywords:** Metabolic dysfunction-associated fatty liver diseases, Mortality, Predictive model, SHAP value, Interpretable machine learning, Hepatology, Machine learning

## Abstract

Emerging evidence links metabolic dysfunction-associated fatty liver disease (MAFLD) with increased all-cause and circulatory system disease (CSD) mortality in adults, yet survival machine learning studies are limited. This study analyzed 4415 NHANES participants with MAFLD to predict mortality using five survival models, and further, the optimal models were selected to identify the most significant predictors of mortality. Machine learning models proved highly effective in prediction. The Gradient Boosted Survival (GBS) model performed best for all-cause mortality, while Extra Survival Trees (EST) excelled for CSD mortality. The Shapley Additive Explanations (SHAP) analyses revealed that the five clinical factors most strongly associated with all-cause mortality were age, gender, platelet count, high-density lipoprotein cholesterol, and smoking status. For CSD mortality, the key factors associated with increased risk were age, blood urea nitrogen, systolic blood pressure, history of heart attack, and gender. Subgroup analyses confirmed GBS and Cox proportional hazard (CoxPH) were optimal for middle-aged and older all-cause mortality, whereas Elastic Net-regularized Cox proportional hazard (CoxNet) was best for older CSD mortality. The findings demonstrate that survival machine learning models effectively predict mortality risk in MAFLD patients. Integrating these models with permutation importance and SHAP provides transparent insights into individual risk profiles, enabling clinicians to clearly interpret how key variables contribute to predictions and improve risk stratification.

**Supplementary Information:**

The online version contains supplementary material available at 10.1038/s41598-025-26729-z.

## Introduction

Metabolic dysfunction-associated fatty liver disease (MAFLD), a clinical entity formally proposed by international experts through the European Liver Patient’s Association in 2020, is characterized by the presence of liver fat deposition along with obesity, diabetes, or combined metabolic dysfunction^1,2^. This novel classification represents a paradigm shift from the traditional non-alcoholic fatty liver disease (NAFLD) framework by establishing positive diagnostic criteria that recognize the multifactorial pathogenesis of metabolic liver disease, independent of significant alcohol intake and concomitant viral hepatitis infection^3,4^. Recent studies reported that the prevalence of MAFLD was more than 30% among the adult population in both the United States and China, and the globally estimated prevalence among overweight or obese adult individuals was approximately 50% ^5–8^. The clinical significance of MAFLD extends beyond hepatology, as longitudinal cohort studies have established its independent association with accelerated progression to cirrhosis, hepatocellular carcinoma incidence, and extrahepatic manifestations, including cardiovascular events and cerebrovascular accidents^1,9–11^. This multisystem involvement, coupled with the high prevalence across metabolic risk strata, positions MAFLD as a critical determinant of global healthcare expenditure and population health outcomes.

Emerging epidemiological evidence has established MAFLD as a multisystem disorder independently associated with progressive hepatic deterioration and elevated risks of all-cause mortality and cardiovascular-specific mortality in adult populations^11–13^. Multicenter cohort studies have quantified these associations, demonstrating adjusted hazard ratios of 1.18 for all-cause mortality in the US adults and 1.14 for cardiovascular mortality in the Korean adults^14,15^. Noteworthy, even when compared with patients with NAFLD, some researchers also found that patients with MAFLD were more likely to suffer from all-cause and cardiovascular mortality^4,13^. Mechanistic investigations identify multifactorial determinants underlying MAFLD-associated mortality, including demographic susceptibility and lifestyle modifiers. Subgroup analysis based on the NHANES cohort revealed differential mortality patterns, with significantly elevated risks observed in MAFLD patients aged ≥ 65 years, non-Hispanic Whites, and paradoxically, those without baseline diabetes^14^. Longitudinal analysis of the Korean Urban Rural Elderly cohort confirmed the independent mortality risk conferred by metabolic abnormalities, persisting after adjustment for hepatic steatosis severity and cardiometabolic medications^16^. Contrastingly, Huang et al.^17^ reported that the level of participation in physical activity (PA) was inversely correlated with all-cause mortality. Moreover, gender, married status, smoking, income level, and the combination of chronic diseases were important factors associated with mortality in patients with fatty liver diseases^18–20^.

Notably, previous studies have demonstrated that several biomarkers were associated with mortality risk in patients with fatty liver diseases. Research conducted by Cheng et al.^18^ demonstrated that higher body mass index (BMI) was a key mortality risk factor for both NAFLD and MAFLD. Another lecture studying the presence of unique phenotypes in patients with NAFLD revealed that the Anthro-SBP (systolic blood pressure)-Glucose phenotype, referring to older individuals with higher BMI, SBP, and fasting blood glucose, had a higher all-cause mortality risk compared to the average phenotype, whereas the lipid-liver phenotype, referring to individuals with higher level of total cholesterol (TC), triglyceride, aspartate aminotransferase (AST), alanine aminotransferase (ALT), and gamma-glutamyl transferase (GGT), was not associated with higher all-cause mortality^21^. In contrast, other studies demonstrated that elevated triglyceride, AST, ALT, and GGT were positively associated with increased mortality risk in patients with MAFLD or MASLD (referring to metabolic dysfunction-associated steatotic liver disease), whereas elevated TC was negatively associated with increased mortality risk^22,23^. These findings reveal that current evidence remains inconsistent regarding the associations of TC, triglyceride, AST, ALT, and GGT with mortality risk, and further investigation to elucidate the underlying mechanisms and clinical implications of these biomarkers is needed. Additionally, previous studies also found that other biomarkers, including diastolic blood pressure (DBP), platelets, total bilirubin, high-density lipoprotein cholesterol (HDL-C), glycohemoglobin (HbA1c), albumin, creatinine, uric acid, and blood urea nitrogen (BUN), were significant predictors associated with all-cause and circulatory system disease (CSD) mortality in patients with NAFLD, MAFLD, or MASLD^22,24–29^.

As an advanced computational paradigm, machine learning utilizes statistical learning algorithms to analyze high-dimensional datasets with complex variable interactions, enabling robust inference of relationships between multidimensional patient characteristics and longitudinal health outcomes^30^. Traditional machine learning models, such as XGBoost, random forest, and LASSO, have been applied across various medical fields to medical image interpretation, diagnostic accuracy enhancement, and disease risk stratification^31–33^. However, for constructing mortality prediction models in time-to-event tasks, survival machine learning is more advantageous than traditional machine learning due to its capacity to handle censored data, predict survival probabilities, estimate hazard and survival functions, and manage time-varying covariates. These capabilities yield more accurate and interpretable predictions in areas where time-to-event outcomes are critical, such as healthcare, customer churn prediction, and predictive maintenance.

In the present investigation, we used a nationally representative cohort of the National Health and Nutrition Examination Survey (NHANES) from the United States to establish the optimal risk prediction models for all-cause and CSD mortality using interpretable survival machine learning methods based on clinical variables among the US middle-aged and older adults. Further, we explored the impact of clinical variables on model construction and identified which clinical variables have strong contributions to the models using the Shapley Additive Explanations (SHAP) framework.

## Methods

### Study population

This study constitutes a secondary analysis utilizing data from the NHANES spanning 1999–2020. NHANES represents a nationally representative cross-sectional survey with longitudinal mortality follow-up, designed to monitor health status and nutritional patterns in the US population through integrated demographic interviews, standardized physical examinations, and laboratory assessments. Administered by the National Center for Health Statistics (NCHS) under the Centers for Disease Control and Prevention (CDC), the survey employs a multistage probability sampling design to select non-institutionalized US civilians, with data collected biennially through in-person household interviews and mobile examination center visits. All the data were publicly available on the website^34^.

**Fig. 1:**
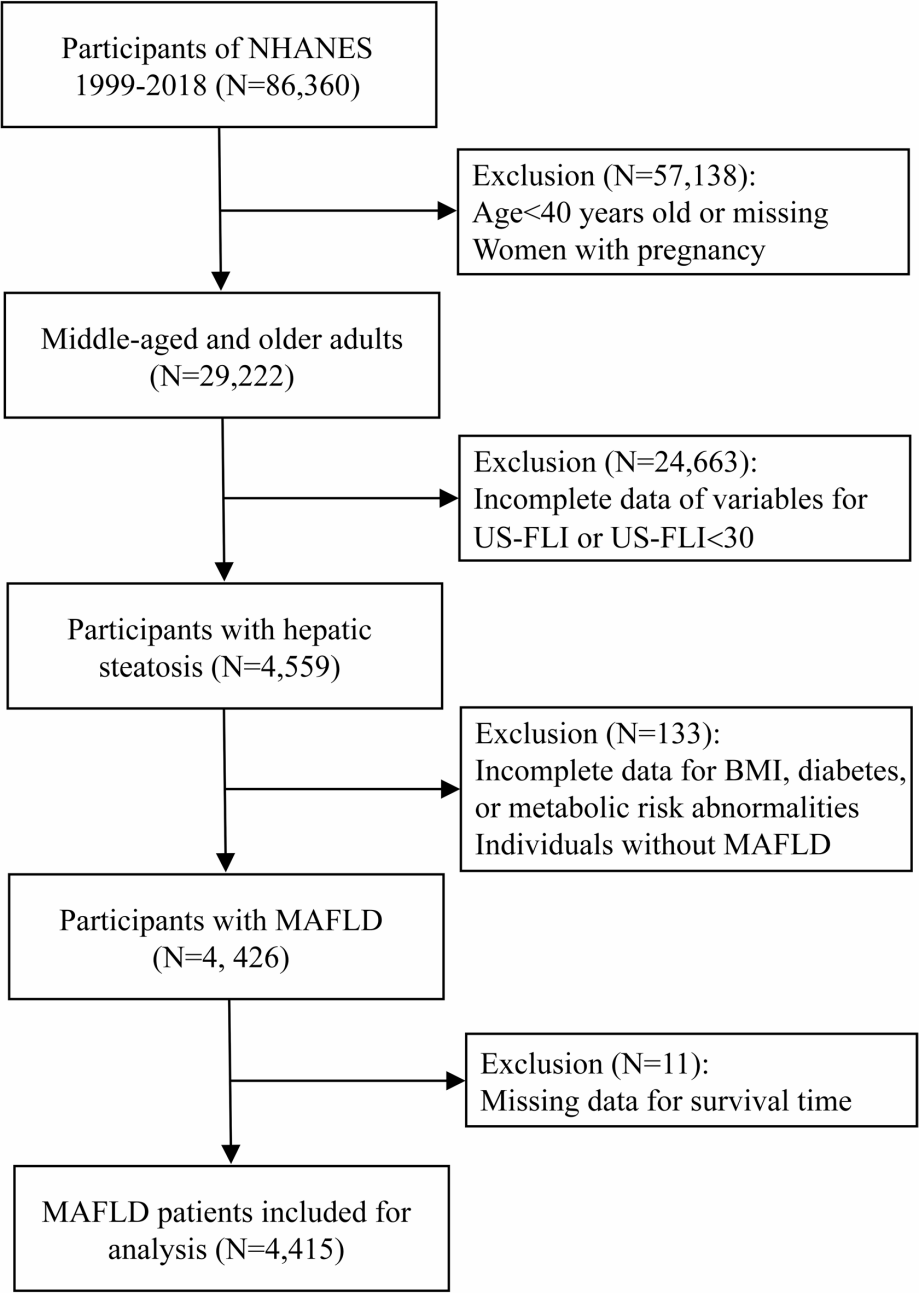
The flowchart for participant selection.

A total of 86,360 individuals were initially enrolled. Mortality outcomes were ascertained through linkage with the National Death Index (NDI), with vital status serving as the primary endpoint. Exclusion criteria included: (1) participants aged < 40 years; (2) incomplete data on core metabolic parameters of age, waist circumference, fasting glucose, insulin, gamma-glutamyl transferase (GGT), and body mass index (BMI); (3) pregnant women. After applying these exclusion parameters, 4415 eligible subjects comprised the analytical cohort (Fig. 1).

### MAFLD definition

MAFLD diagnosis was defined according to the international consensus criteria proposed by an expert panel, requiring the presence of hepatic steatosis plus at least one of three metabolic risk components^1^. Hepatic steatosis was determined using the US Fatty Liver Index (US-FLI) with a validated cutoff score ≥ 30^35^. The US-FLI calculation formula was implemented as follows:

$${\text{US - FLI}} = \:\frac{{e^{\begin{gathered} - 0.8073 \times \:non - Hispannic\:black + 0.3458 \times \: \hfill \\ Mexican - Ameican + 0.0093 \times \:age \hfill \\ + 0.6151 \times \:log\_e\left( {gamma - glutamyl\:transferase} \right) \hfill \\ + 0.0249 \times \:wasit\:circumstance + 1.1792 \times \:log\_e\left( {insulin} \right) \hfill \\ + 0.8242 \times \:log\_e\left( {glucose} \right) - 14.7812 \hfill \\ \end{gathered} } }}{{1 + e^{\begin{gathered} - 0.8073 \times \:non - Hispannic\:black + 0.3458 \hfill \\ \times \:Mexican - Ameican + 0.0093 \hfill \\ \times \:age + 0.6151 \times \:log\_e\left( {gamma - glutamyl\:transferase} \right) \hfill \\ + 0.0249 \times \:wasit\:circumstance + 1.1792 \hfill \\ \times \:log\_e\left( {insulin} \right) + 0.8242 \times \:log\_e\left( {glucose} \right) - 14.7812 \hfill \\ \end{gathered} } }} \times \:100$$

The other three features included: (ⅰ) overweight or obesity (defined as BMI ≥ 25 kg m^2^ in Caucasians or BMI ≥ 23 kg m^2^ in Asians); (ⅱ) Type 2 diabetes mellitus (a self-reported history or HbA1c ≥ 6.5%); (ⅲ) metabolic dysregulation for lean/normal weight (presence of at least two metabolic risk abnormalities; defined as BMI < 25 kg/m^2^ in Caucasians or BMI < 23 kg/m^2^ in Asians): (1) Waist circumference ≥ 102/88 cm in Caucasian men and women (or ≥ 90/80 cm in Asian men and women), (2) Blood pressure ≥ 130/85 mmHg or specific drug treatment, (3) Plasma triglycerides ≥ 150 mg/dl (≥ 1.70 mmol/L) or specific drug treatment, (4) Plasma HDL-C < 40 mg/dl (< 1.0 mmol/L) for men and < 50 mg/dl (< 1.3 mmol/L) for women or specific drug treatment, (5) Prediabetes (i.e., fasting glucose levels 100 to 125 mg/dl {5.6 to 6.9 mmol/L}, or 2-hour post-load glucose levels 140 to 199 mg/dl {7.8 to 11.0 mmol} or HbAlc 5.7% to 6.4% {39 to 47 mmol/mol}), (6) Homeostasis model assessment of insulin resistance (HOMA-IR) score ≥ 2.5 ^36^, (7) Plasma high-sensitivity C-reactive protein level ≥ 2 mg/L.

### Mortality ascertainment

Mortality ascertainment (updated through December 31, 2019) was obtained via restricted-access linkage with the NCHS^37^, deterministically matched to NHANES exposure data through participants’ unique identifiers. Follow-up duration was computed in person-months, commencing at the baseline examination date and extending until death occurrence or administrative censoring on December 31, 2019. Mortality outcomes were stratified according to the International Classification of Diseases 10th Revision (ICD-10) into two categories: all-cause mortality and CSD mortality (I00-I99).

### Covariates

Demographic characteristics included age, gender (male/female), ethnicity (Mexican American/Other Hispanic/Non-Hispanic White/Non-Hispanic Black/Other Race-Including Multi-Racial), level of education (below high school/high school/above high school), married status (married or living with partner/widowed or divorced or separated/never married), income level(low income for income-poverty ratio < 1.3/middle income for income-poverty ratio ≥ 1.3 and < 3.5, high income for income-poverty ratio ≥ 3.5) ^38^.

Health-related covariates consisted of smoking status (non-smoker/former-smoker/current-smoker), alcohol assumption (alcoholism/non-alcoholism), and BMI (‌categorized as < 18.5 kg/m^2^, 18.5–25 kg/m^2^, 25–30 kg/m^2^, or ≥ 30 kg/m^2^), chronic diseases (yes/no), PA (physically active/ physically inactive), infected with hepatitis B virus (yes/no), infected with hepatitis C virus (yes/no), sleep (hours), waist circumference (cm), SBP (mmHg), DBP (mmHg), pulse (times per min), platelet (1000 cells/uL), ALT (U/L), AST (U/L), total bilirubin (mg/dL), GGT (U/L), albumin (g/L), triglyceride (mg/dL), TC (mg/dL), HDL-C (mg/dL), low-density lipoprotein cholesterol (LDL-C, mg/dL), HbA1c (%), fasting blood glucose (mg/dL), fasting insulin (pmol/L), creatinine (mg/dL), uric acid (mg/dL), BUN (mg/dL). For different smoking status, the non-smokers were those having smoked less than 100 cigarettes in their lifetime; the former-smokers were those having smoked more than 100 cigarettes in their lifetime but quit smoking, and the current-smokers were those having smoked more than 100 cigarettes in their lifetime and currently smoking^39,40^. With respect to the alcohol assumption, alcoholism referred to those who had more than 2 drinks per day for men or more than 1 drink per day for women^41^. Chronic diseases included hypertension, congestive heart failure, coronary heart disease, angina, heart attack, stroke, diabetes, kidney disease, asthma, arthritis, thyroid problems, and cancer. PA was categorized according to the 2018 Physical Activity Guidelines, which define adults who engage in ≥ 150 minutes per week of moderate-intensity PA, 75 minutes per week of vigorous-intensity PA, or an equivalent combination as meeting the guidelines. We categorized PA as either “physically inactive (not meeting the guidelines)” or “physically active (meeting the guidelines) ^38,42^.

### Statistical analysis

#### Baseline data analysis

Statistical preprocessing was conducted using Stata SE 15.0 and R 4.3.3, implementing rigorous multiple imputation for variables with < 10% missingness after excluding cases with incomplete survival status, follow-up duration, or US-FLI calculation parameters. Continuous variables were summarized as mean ± SD or median (interquartile range, IQR), and categorical variables as frequency (%). Group comparisons employed Student’s t-test or Mann–Whitney U for continuous measures and chi-square or Fisher’s exact tests for categorical distributions. All analyses employed two-tailed tests with *p* = 0.05 significance threshold.

#### Feature selection and model development

To assess the model’s robustness and predictive performance, a hold-out validation approach was adopted, allowing for an objective and independent evaluation. The entire dataset was randomly divided into an 80% (*N* = 3532) training set and a 20% (*N* = 883) validation set to ensure consistent comparability across methodological approaches. Feature selection was conducted in the training set using LASSO regression, and covariates with a non-zero coefficient in the LASSO regression were entered into the survival models. Additionally, patients were stratified into middle-aged (40–59 years) and older (≥ 60 years) subgroups in the subsequent age-stratified analyses, which further supported refined risk assessment and personalized decision-making.

For mortality prediction modeling, we implemented five survival machine learning architectures via scikit-survival 0.23.0 in Python 3.12, including Cox proportional hazard (CoxPH), Elastic Net-regularized Cox proportional hazard (CoxNet), Random Survival Forest (RSF), Gradient Boosted Survival (GBS), and Extra Survival Trees (EST). CoxPH is the standard approach for survival analysis. As a semi-parametric method, it estimates the risk of individual patients experiencing the event of interest by integrating a population-level baseline hazard function with individual-specific static predictive covariates^43^. CoxNet is a penalized regression method for Cox models that, in contrast to the traditional CoxPH model, effectively handles the dataset with high dimensions and variable collinearity compared with the traditional Cox algorithm^44^. RSF is an extension of the random forest algorithm that operates without relying on stringent prior assumptions by employing random feature selection and ensemble averaging, thereby effectively capturing complex nonlinear covariate effects^43^. GBS is an adaptation of the gradient boosting machine specifically designed for survival analysis, capable of capturing complex feature-time interactions and non-linear associations independent of the proportional hazards assumption^45^. EST extends the extremely randomized trees model through the integration of enhanced randomization mechanisms and recursive partitioning to identify homogeneous risk subgroups based on feature splits, thereby yielding a tree structure capable of predicting survival outcomes for new observations^46^. A growing body of research has employed these survival machine learning models, consistently reporting strong predictive accuracy^43–46^.

As the event of death is rare but significant in survival analysis, it may lead to a degradation in the performance of the predictive models. Indeed, we used the SMOTE method, also named the “artificial minority oversampling method”, to oversample the deaths to increase its sample size, tackling an imbalanced dataset in the train set^47^. Moreover, to identify optimal hyperparameters for each algorithm, we performed hyperparameter optimization using the scikit-survival library. This involved an exhaustive grid search strategy (GridSearchCV function) with five-fold cross-validation applied to the training set, aiming to maximize model performance from a predefined parameter space^44^.

#### Model validation

Model validation emphasized two principal aspects: discrimination, quantified by the time-dependent AUC (tAUC) and Harrell’s C-index, which assess the model’s ability to differentiate between outcomes; and calibration, evaluated using the integrated Brier score (IBS), which reflects the concordance between predicted probabilities and actual observed outcomes. The tAUC primarily evaluates the discriminatory power of a model, which reflects its ability to distinguish between patients who will experience an event and those who will not within a specified time horizon^48^. A higher tAUC value indicates enhanced capability of the model in early identification of high-risk individuals. The C-index assesses the performance of a model by quantifying the proportion of concordant sample pairs, reflecting the advantage of handling censored data^49^. A higher C-index indicates a model’s accuracy in predicting the order of events. Moreover, the IBS quantifies the discrepancy between predicted probabilities and observed outcomes across the event range^50^. A lower IBS value reflects superior calibration of the model’s predictions to actual events, thereby strengthening its clinical applicability.

#### Model interpretation

The SHAP framework, widely used in clinical research to analyze non-linear relationships and complex interactions in high-dimensional data, was employed to estimate feature importance^30^. It assigns an importance value to each feature and provides a visual interpretation of the optimal model, based on cooperative game theory. This study identified important predictors of all-cause and CSD mortality, and further assessed the associations between variables and mortality using SHAP from the final model.

## Results

### Baseline characteristics

The analytical cohort comprised 4415 participants with a median follow-up of 87 months (IQR 47–135), demonstrating a mean age of 60.89 ± 11.80 years and 43.19% male predominance (Table 1). Through follow-up termination, 941 (23.31%) all-cause deaths occurred, including 299 (6.77%) CSD deaths. Comparative analysis revealed significant demographic disparities: decedents in all-cause mortality analysis were older (69.60 ± 10.43 vs. 58.53 ± 11.02 years) with a higher female proportion (64.61% vs. 56.69%) compared to survivors. Multivariable assessment identified other clinical parameters showing intergroup differences (*p* < 0.05), including ethnicity, education level, married status, alcohol assumption, chronic diseases except for asthma and thyroid problems, PA, smoking status, income level, BMI, ALT, creatinine, uric acid, BUN, total bilirubin, GGT, waist circumference, SBP, DBP, platelet, HbAlc, fasting blood glucose, HDL-C, LDL-C. CSD mortality analysis mirrored age (70.89 ± 10.51 vs. 60.17 ± 11.56 years) and gender (64.55% vs. 56.24% female) disparities, whereas different from the all-cause mortality analysis, variables including alcohol assumption, morbidity of cancer, PA, BMI, GGT, waist circumference, platelet, and LDL-C were insignificantly different between participants who were dead and alive in the CSD mortality analysis. Further comparative statistics in subgroups across all-cause and CSD mortality cohorts are detailed in Supplementary Tables 1–3.

**Table 1:** Baseline characteristics for all-cause mortality and CSD mortality among all participants.

Variables	Total (*n* = 4415)	All-cause mortality			CSD mortality		
		Alive (*n* = 3474)	Dead (*n* = 941)	*P*	Alive (*n* = 4116)	Dead (*n* = 299)	*P*
Gender (n, %)				<0.001			0.005
Male	1907 (43.19%)	1574 (45.31%)	333 (35.39%)		1801 (43.76%)	106 (35.45%)	
Female	2508 (56.81%)	1900 (54.69%)	608 (64.61%)		2315 (56.24%)	193 (64.55%)	
Ethnicity (n, %)				<0.001			<0.001
Mexican American	1115 (25.25%)	910 (26.19%)	205 (21.79%)		1050 (25.51%)	65 (21.74%)	
Other Hispanic	483 (10.94%)	433 (12.46%)	50 (5.31%)		467 (11.35%)	16 (5.35%)	
Non-Hispanic White	2010 (45.53%)	1448 (41.68%)	562 (59.72%)		1835 (44.58%)	175 (58.53%)	
Non-Hispanic Black	484 (10.96%)	389 (11.20%)	95 (10.10%)		451 (10.96%)	33 (11.04%)	
Other Race-Including Multi-Racial	323 (7.32%)	294 (8.47%)	29 (3.08%)		313 (7.60%)	10 (3.34%)	
Education level (n, %)				<0.001			<0.001
Below high school	1555 (35.22%)	1141 (32.84%)	414 (44.00%)		1423 (34.57%)	132 (44.15%)	
High school	946 (21.43%)	725 (20.87%)	221 (23.48%)		874 (21.23%)	75 (24.08%)	
Above high school	1914 (43.35%)	1608 (46.29%)	306 (32.52%)		1819 (44.20%)	95 (31.77%)	
Married status (n, %)				<0.001			<0.001
Married/ Living with Partner	2870 (65.00%)	2334 (67.18%)	536 (56.96%)		2710 (65.84%)	160 (53.51%)	
Widowed/ Divorced/ Separated	1236 (28.00%)	882 (25.39%)	354 (37.62%)		1116 (27.11%)	120 (40.13%)	
Never married	309 (7.00%)	258 (7.43%)	51 (5.42%)		290 (7.05%)	19 (6.36%)	
Income level (n, %)				<0.001			0.005
Low income	1333 (30.19%)	1007 (28.99%)	326 (34.64%)		1235 (30.00%)	98 (32.77%)	
Middle income	1868 (42.31%)	1419 (40.85%)	449 (47.72%)		1725 (41.91%)	143 (47.83%)	
High income	1214 (27.50%)	1048 (30.16%)	166 (17.64%)		1156 (28.09%)	58 (19.40%)	
Smoke status (n, %)				<0.001			0.024
Non-smoker	2070 (46.89%)	1720 (49.51%)	350 (37.19%)		1945 (47.25%)	125 (41.80%)	
Former-smoker	1596 (36.15%)	1170 (33.68%)	426 (45.27%)		1466 (35.62%)	130 (43.48%)	
Current-smoker	749 (16.96%)	584 (16.81%)	165 (17.54%)		705 (17.13%)	44 (14.72%)	
Alcohol assumption (n, %)				0.040			0.198
No	4085 (92.53%)	3229 (92.95%)	856 (90.97%)		3814 (92.66%)	271 (90.64%)	
Yes	330 (7.47%)	245 (7.05%)	85 (9.23%)		302 (7.34%)	28 (9.36%)	
BMI (n, %)				<0.001			0.273
<18.5 kg/m^2^	5 (0.11%)	1 (0.03%)	4 (0.43%)		4 (0.10%)	1 (0.33%)	
18.5–25 kg/m^2^	292 (6.62%)	189 (5.43%)	103 (10.94%)		268 (6.51%)	24 (8.03%)	
25–30 kg/m^2^	1483 (33.59%)	1107 (31.87%)	376 (39.96%)		1375 (33.41%)	108 (36.12%)	
≥30 kg/m^2^	2635 (59.68%)	2177 (62.67%)	458 (48.67%)		2469 (59.98%)	166 (55.52%)	
Chronic diseases (n, %)							
Hypertension	2495 (56.51%)	1884 (54.23%)	611 (64.93%)	<0.001	2287 (55.56%)	208 (69.57%)	<0.001
Congestive heart failure	269 (6.09%)	147 (4.23%)	122 (12.96%)	<0.001	235 (5.71%)	34 (11.37%)	<0.001
Coronary heart disease	354 (8.02%)	222 (6.39%)	132 (14.03%)	<0.001	305 (7.41%)	49 (16.39%)	<0.001
Angina	229 (5.19%)	129 (3.71%)	100 (10.63%)	<0.001	191 (4.64%)	38 (12.71%)	<0.001
Heart attack	349 (7.90%)	210 (6.04%)	139 (14.77%)	<0.001	302 (7.34%)	47 (15.72%)	<0.001
Stroke	240 (5.44%)	137 (3.94%)	103 (10.95%)	<0.001	205 (4.98%)	35 (11.71%)	<0.001
Diabetes	1151 (26.07%)	869 (25.01%)	282 (29.97%)	0.002	1050 (25.51%)	101 (33.78%)	0.002
Kidney disease	204 (4.62%)	143 (4.12%)	61 (6.48%)	0.002	181 (4.40%)	23 (7.69%)	0.009
Asthma	588 (13.32%)	467 (13.44%)	121 (12.86%)	0.640	549 (13.34%)	39 (13.04%)	0.885
Arthritis	1860 (42.13%)	1375 (39.58%)	485 (51.54%)	<0.001	1708 (41.50%)	152 (50.84%)	0.002
Thyroid problem	571 (12.93%)	465 (13.39%)	106 (11.26%)	0.086	539 (13.10%)	32 (10.70%)	0.234
Cancer	582 (13.18%)	392 (11.28%)	190 (20.19%)	<0.001	533 (12.95%)	49 (16.39%)	0.090
Hepatitis B (n, %)	21 (0.48%)	17 (0.49%)	4 (0.43%)	0.799	21 (0.51%)	0 (0.00%)	0.216
Hepatitis C (n, %)	126 (2.85%)	96 (2.76%)	30 (3.19%)	0.488	121 (2.94%)	5 (1.67%)	0.204
Physical activity (n, %)	2327 (52.71%)	1885 (54.26%)	442 (46.97%)	<0.001	2185 (53.09%)	142 (47.49%)	0.061
Age (yrs)	60.89 ± 11.80	58.53 ± 11.02	69.60 ± 10.43	<0.001	60.17 ± 11.56	70.89 ± 10.51	<0.001
Sleep (hours)	7.23 ± 1.44	7.22 ± 1.46	7.26 ± 1.38	0.369	7.21 ± 1.45	7.39 ± 1.35	0.044
Waist circumference (cm)	109.66 ± 13.23	109.96 ± 13.27	108.57 ± 13.05	0.004	109.68 ± 13.27	109.38 ± 12.65	0.699
SBP (mmHg)	130.63 ± 18.53	129.21 ± 17.36	135.89 ± 21.56	<0.001	130.05 ± 18.13	138.64 ± 21.87	<0.001
DBP (mmHg)	71.96 ± 12.43	73.07 ± 11.83	67.86 ± 13.65	<0.001	72.37 ± 12.14	66.38 ± 14.80	<0.001
Pulse (times per minute)	71.89 ± 12.18	71.73 ± 11.83	72.49 ± 13.37	0.088	71.91 ± 12.13	71.65 ± 12.80	0.719
Platelet (1000 cells/uL)	239.77 ± 66.58	241.52 ± 65.60	233.33 ± 69.74	0.001	240.17 ± 66.33	234.34 ± 66.72	0.144
ALT (U/L)	30.28 ± 25.10	31.06 ± 25.69	27.40 ± 22.57	<0.001	30.53 ± 25.02	26.85 ± 25.94	0.014
AST (U/L)	28.22 ± 23.61	28.05 ± 24.58	28.85 ± 19.57	0.358	28.25 ± 23.72	27.86 ± 21.99	0.783
Total bilirubin (mg/dL)	0.68 ± 0.30	0.66 ± 0.29	0.74 ± 0.32	<0.001	0.67 ± 0.30	0.71 ± 0.29	0.033
GGT (U/L)	46.25 ± 67.70	45.16 ± 65.20	50.27 ± 67.41	0.034	46.19 ± 65.52	47.12 ± 68.31	0.834
Albumin (g/L)	41.79 ± 3.29	41.81 ± 3.24	41.71 ± 3.47	0.378	41.79 ± 3.28	41.77 ± 3.42	0.893
Triglyceride (mg/dL)	170.11 ± 117.31	168.72 ± 119.32	175.23 ± 109.50	0.131	169.38 ± 117.79	180.18 ± 110.25	0.124
TC (mg/dL)	196.97 ± 43.91	197.47 ± 43.63	195.15 ± 43.91	0.151	196.96 ± 43.73	197.10 ± 46.46	0.958
HDL-C (mg/dL)	44.84 ± 16.57	45.86 ± 14.83	41.05 ± 21.42	<0.001	45.10 ± 16.15	41.19 ± 21.30	<0.001
LDL-C (mg/dL)	115.61 ± 37.47	116.59 ± 37.31	112.01 ± 37.89	0.001	115.82 ± 37.45	112.75 ± 37.71	0.171
HbA1c (%)	6.29 ± 1.42	6.27 ± 1.38	6.38 ± 1.56	0.030	6.27 ± 1.39	6.61 ± 1.82	<0.001
Fasting Glu (mg/dL)	128.42 ± 48.48	126.87 ± 46.28	134.12 ± 55.49	<0.001	125.52 ± 47.10	140.71 ± 63.31	<0.001
Fasting insulin (pmol/L)	135.55 ± 158.24	135.89 ± 164.04	134.30 ± 134.76	0.784	135.64 ± 161.19	134.29 ± 109.94	0.887
Creatinine (mg/dL)	0.91 ± 0.41	0.88 ± 0.33	1.03 ± 0.62	<0.001	0.90 ± 0.40	1.04 ± 0.50	<0.001
Uric acid (mg/dL)	6.00 ± 1.47	5.94 ± 1.43	6.24 ± 1.61	<0.001	5.97 ± 1.46	6.40 ± 1.63	<0.001
BUN (mg/dL)	15.25 ± 6.44	14.60 ± 5.65	17.66 ± 8.33	<0.001	15.00 ± 6.16	18.70 ± 8.80	<0.001

### Model construction and selection

Tables 2 and 3 present the comparative performance evaluation of survival models for all-cause and CSD mortality prediction across overall and age-stratified cohorts. Model discrimination was quantified through tAUC and C-index, while calibration was assessed via IBS, with comprehensive benchmarking conducted across five machine learning architectures.

**Table 2:** Performance of the models for predicting all-cause and CSD mortality in overall analyses.

Group	Index	CoxPH	RSF	CoxNet	EST	GBS
All-cause mortality	tAUC	0.845	0.852	0.845	0.835	0.854
	C-index	0.797	0.804	0.796	0.794	0.815
	IBS	0.102	0.105	0.101	0.114	0.100
CSD mortality	tAUC	0.855	0.866	0.856	0.879	0.837
	C-index	0.814	0.812	0.815	0.824	0.787
	IBS	0.056	0.058	0.056	0.060	0.060

**Table 3:** Performance of the models for predicting all-cause and CSD mortality in subgroup analyses.

Group	Index	CoxPH	RSF	CoxNet	EST	GBS
All-cause mortality for middle-aged adults	tAUC	0.803	0.785	0.803	0.821	0.845
	C-index	0.755	0.773	0.757	0.784	0.800
	IBS	0.075	0.080	0.074	0.070	0.067
All-cause mortality for older adults	tAUC	0.826	0.819	0.829	0.805	0.825
	C-index	0.777	0.785	0.779	0.768	0.781
	IBS	0.134	0.142	0.133	0.151	0.138
CSD mortality for older adults	tAUC	0.821	0.754	0.815	0.778	0.762
	C-index	0.776	0.730	0.774	0.747	0.728
	IBS	0.084	0.096	0.085	0.096	0.094

**Fig. 2:**
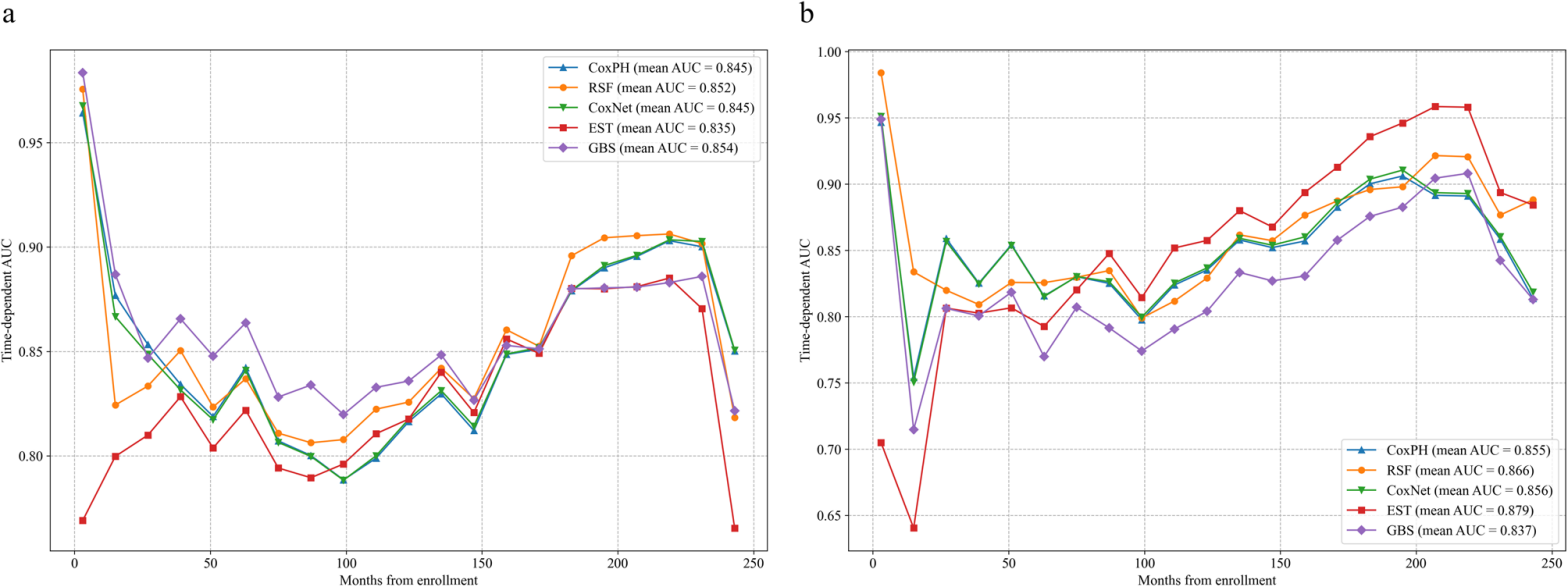
Time-dependent AUC of different models for predicting mortality in the overall analyses. (**a**) Models with all-cause mortality, (**b**) models with CSD mortality.

In the overall cohort analysis, the GBS model demonstrated superior discriminative capacity for all-cause mortality prediction (tAUC = 0.854, C-index = 0.815) with optimal calibration (IBS = 0.100), outperforming other algorithms (Table 2; Fig. 2a). Conversely, EST emerged as the optimal CSD mortality predictor (tAUC = 0.879, C-index = 0.824) despite suboptimal calibration (IBS = 0.060) (Table 2; Fig. 2b).

**Fig. 3:**
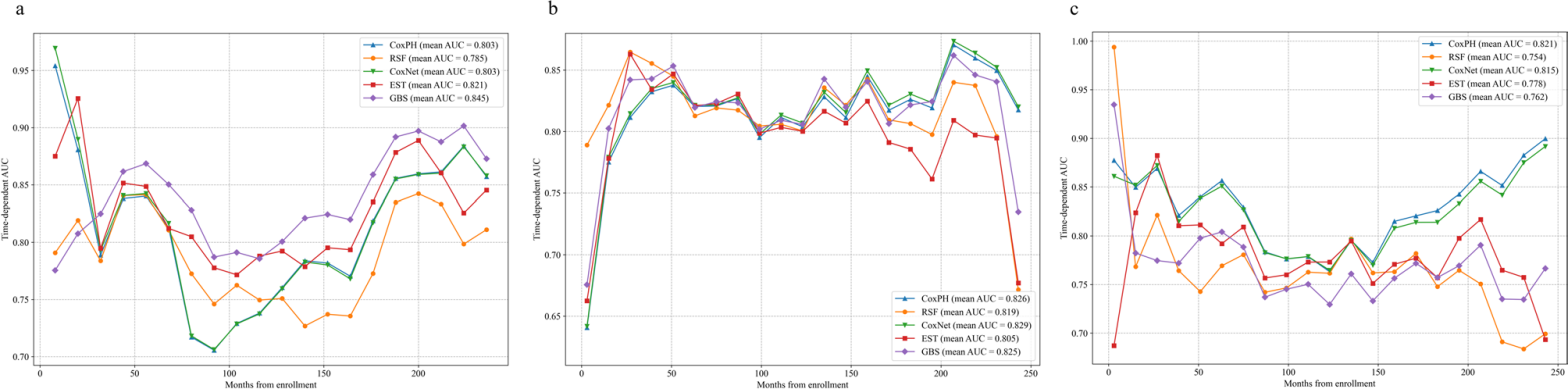
Time-dependent AUC of different models for predicting mortality in the subgroup analyses. (**a**) Models with all-cause mortality for middle-aged adults, (**b**) models with all-cause mortality for older adults, (**c**) models with CSD mortality for older adults.

Age-stratified analysis revealed distinct optimal models: (1) Middle-aged cohort: GBS maintained prediction supremacy for all-cause mortality (tAUC = 0.845, C-index = 0.800, IBS = 0.067; Table 3; Fig. 3a); (2) Older cohort: CoxNet showed balanced performance for all-cause mortality (tAUC = 0.829, C-index = 0.779, IBS = 0.15; Table 3; Fig. 3b), while CoxPH achieved optimal CSD mortality prediction (tAUC = 0.821, C-index = 0.776, IBS = 0.084; Table 3; Fig. 3c). Notably, CSD mortality modeling in middle-aged adults exhibited technical limitations, with all algorithms failing to generate valid tAUC estimates, precluding further interpretation.

### Permutation importance and visualization by SHAP

**Fig. 4:**
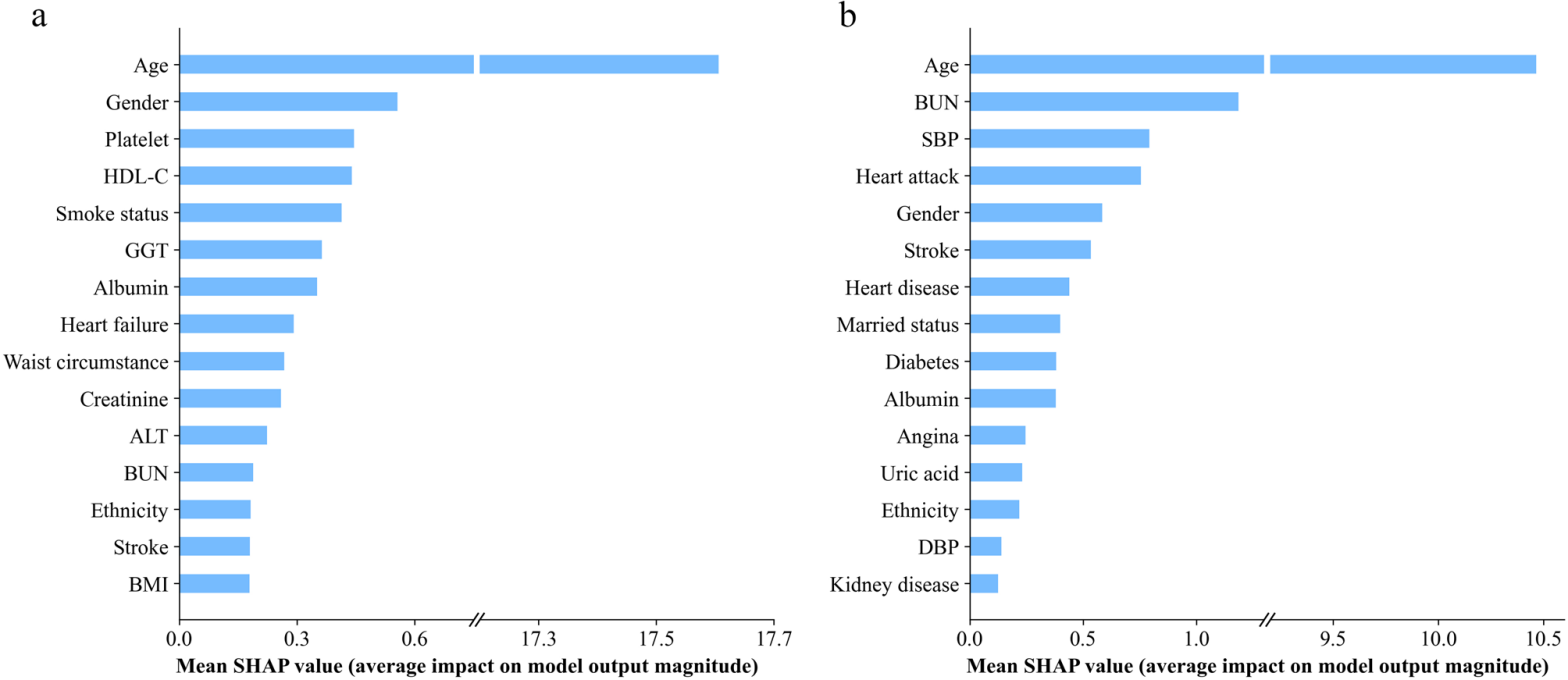
Summary plot of optimal model for prediction models by SHAP in the overall analyses. (**a**) Models with all-cause mortality, (**b**) models with CSD mortality.

Figure 4 delineates the SHAP-derived feature importance rankings across optimal mortality prediction models. Age showed the strongest association with both all-cause (SHAP value 0.176 ± 0.019) and CSD mortality (0.105 ± 0.019) in the overall cohort (Fig. 4a,b). After age, the four most important features associated with all-cause mortality were gender, platelet, HDL-C, and smoking status; but for CSD mortality, they were BUN, SBP, history of heart attack, and gender.

**Fig. 5:**
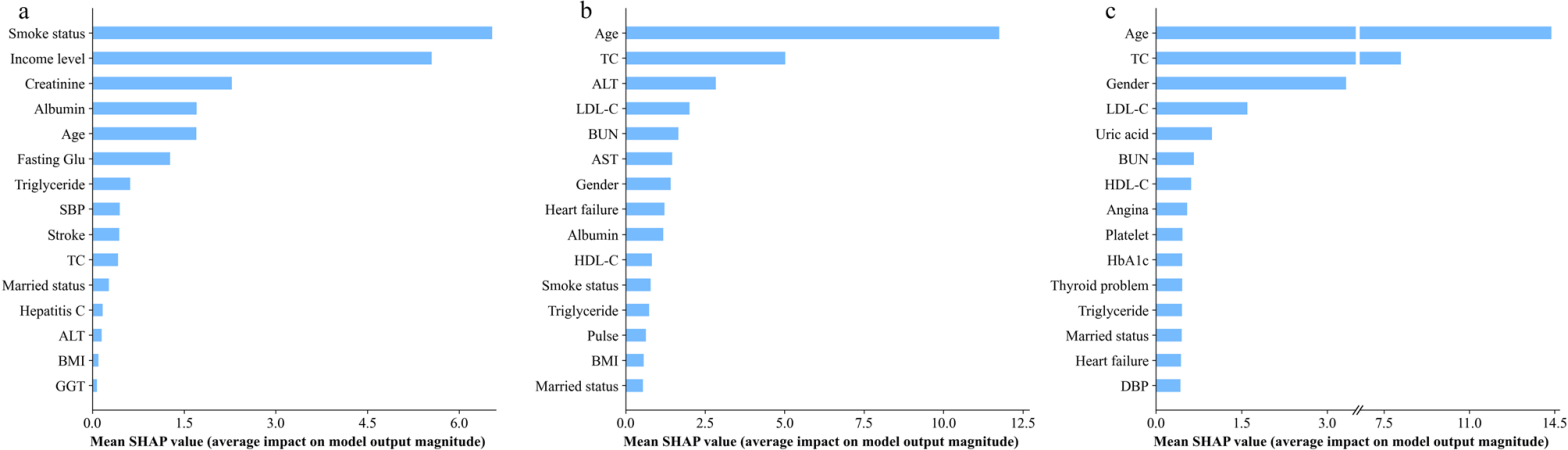
Summary plot of optimal model for prediction models by SHAP in the subgroup analyses. (**a**) Models with all-cause mortality for middle-aged adults, (**b**) models with all-cause mortality for older adults, (**c**) models with CSD mortality for older adults.

Age-stratified SHAP analysis (Fig. 5) revealed divergent patterns: In middle-aged cohorts, smoking status exhibited the strongest association with all-cause mortality prediction (0.065 ± 0.020), while age importance declined to fifth position (0.017 ± 0.019 vs. 0.176 ± 0.019 overall). Conversely, older cohorts retained the highest association for age (all-cause mortality: 0.118 ± 0.021; CSD mortality: 0.143 ± 0.02). Notably, lipid profiles (TC, LDL-C) showed stronger associations in older adult models compared to middle-aged counterparts. Technical limitations precluded CSD mortality modeling in middle-aged adults due to insufficient event rates for tAUC computation.

## Discussion

The present study, utilizing population-based cohort data from the NHANES dataset, demonstrated that both GBS and EST models exhibited superior predictive performance for all-cause and CSD mortality, respectively. Subsequent analyses revealed that the five clinical factors most strongly associated with all-cause mortality were age, gender, platelet count, HDL-C levels, and smoking status. For CSD mortality, the key factors associated with increased risk were age, BUN, SBP, history of heart attack, and gender. Subgroup analyses identified GBS and CoxPH models as optimal for all-cause mortality prediction, while the CoxNet model showed optimal performance for CSD mortality prediction.

Nowadays, disease predictive models, such as the Framingham risk score and the ASCVD risk estimator for cardiovascular disease risk, have been developed and used widely. However, their uses in mortality risk prediction were limited. For example, de Ruijter and colleagues reported that in very old age, classic risk factors as included in the Framingham risk score no longer predicted 5-year cardiovascular mortality in people with no history of cardiovascular disease^51^. As machine learning model develops, mortality prediction models have been established and used in common diseases like cardiovascular diseases, and acute and chronic kidney disease^52–54^, whereas in MAFLD, their usage remains understudied. To date, only two predictive models have been developed: Decraecker et al.^55^ reported a Cox model (C-index 0.8–0.9) incorporating hepatic parameters, while Bonfiglio et al.^22^ developed a model (C-index 0.84–0.85) integrating demographic and metabolic variables. Notably, survival machine learning models, including RSF, CoxPH, CoxNet, and so on, have advantages that traditional machine learning because of their capacity to manage time-varying covariates and capacity to handle censored data, and have been used in predicting all-cause and case-specific mortality in the total population^47,56^. In this study, we demonstrated that four survival machine learning models had the optimal capabilities in all-cause and CSD mortality risk prediction, with the C-index ranging from 0.776 to 0.824. Despite the lack of external validation, we propose that these models hold significant potential for generalization in MAFLD and may demonstrate considerable value in predicting mortality risks associated with other diseases.

In this study, we identified both age and gender as key factors associated with all-cause and CSD mortality in the optimal models for MAFLD. These findings were consistent with previous studies, which have also observed associations of older age and female sex with increased risks of all-cause and CSD mortality in MAFLD patients^14,18,22,57,58^. In a cohort study, Cheng et al.^18^ reported that older age was associated with an increased risk of all-cause mortality in both NAFLD and MAFLD, with multivariable-adjusted HRs of 1.09 and 1.08 per year increase in age, respectively; meanwhile, similar associations were observed between older age and cardiovascular disease mortality in their analyses. Additionally, we conducted subgroup analyses to assess the mortality prediction model across age-stratified groups, and age remained significantly associated with both all-cause and CSD mortality in prediction models for older adults. Based on these findings, we speculated that the optimal models for all-cause and CSD mortality in overall and subgroup analyses might perform as well when they included age as the sole variable, and then conducted further analyses to investigate this possibility. Results showed that in age-only models, the C-index for all-cause mortality and CSD mortality in overall analyses, all-cause mortality in middle-aged and older individuals, and CSD mortality in older adults were 0.750, 0.743, 0.616, 0.708, and 0.743, respectively (Supplementary Tables 4–5; Supplementary Fig. 1–2), and all of them were significantly lower than those in the overall models. These findings indicate that while age is a robust predictor, it does not fully capture the complex risk profile associated with all-cause and CSD mortality in MAFLD. Its predictive capability may stem from its role as a composite marker for declining multiple physiological functions, accumulation of comorbidities, and increased frailty. Nevertheless, to assess mortality risk more comprehensively in MAFLD, age should be integrated with other clinical variables, such as gender, modified lifestyles, and biomarkers. Therefore, age should be incorporated as a core component rather than the sole variable in establishing predictive models for all-cause and CSD mortality in MAFLD. Further model development should integrate age with other key covariates to enhance discriminative ability and clinical utility.

Regarding sex differences in the survival model predictions, it showed that in the overall and subgroup for all-cause mortality and CSD mortality, the proportions of females who died during the follow-up period ranged from 56.24% to 68.35% and were higher than those of males. Moreover, gender remained significantly associated with mortality in prediction models for all-cause and CSD mortality. Nevertheless, findings regarding sex differences in all-cause and CSD mortality were inconsistent. For example, two studies conducted by Decraecker et al.^55^ and Cheng et al.^18^ reported that male sex was associated with an increased risk of mortality, while female sex was correlated with reduced risk among individuals with MAFLD or NAFLD. Conversely, a meta-analysis demonstrated that women had a 1.5-fold higher risk of all-cause mortality and a 2-fold higher risk of cardiovascular disease mortality compared to men, with corresponding OR of 1.65 and 2.12, respectively^57^. Potential reasons for these divergent findings may be due to the differences among the definitions of liver steatosis, racial populations, and follow-up periods.

Other significant variables associated with all-cause and CSD mortality in both overall and subgroup prediction models included several health-related covariates, such as smoking status, SBP, TC, HDL-C, LDL-C, and uric acid. These factors have been linked to increased mortality risks in patients with MAFLD^59–61^. In contrast, key variables, including platelet count, BUN, income level, and creatinine, lack reported correlations with mortality risk in this population; thus, further studies focusing on their potential relationships with clinical outcomes should be conducted.

It is worth noting that several previous studies reported that PA was critical for the outcomes of patients with fatty liver disease. For patients with NAFLD, researchers found that increasing PA, no matter the duration or intensity, was associated with a reduced risk of all-cause and cardiovascular mortality^62–65^. Similar findings were reported in patients with MAFLD, in which researchers revealed that PA level was inversely correlated with all-cause mortality but not correlated with cardiovascular disease mortality^17^. However, in the current study, we found that PA was not associated with mortality in all prediction models. We hypothesize that the observed relationship may stem from the fact that increasing PA effectively improves physical symptoms and is associated with reduced mortality risk. Conversely, a lack of PA may not be significantly associated with increased mortality. Nonetheless, we assert that further research is needed to establish the relationship between PA and MAFLD mortality.

In this study, the SHAP analysis identified age, BUN, TC, SBP, and other key variables as the influential and valuable predictors of mortality in MAFLD patients. These findings offer actionable insights for clinical risk stratification and personalized management. For example, age is a non-modifiable yet powerful indicator, so older patients with MAFLD should be prioritized for more frequent monitoring and comprehensive geriatric assessment. Elevated BUN may indicate that MAFLD patients have impaired renal function or dehydration, and they need renal protection strategies and fluid management. Increased TC levels emphasize the importance of lipid metabolic health and may call for cholesterol-lowering treatments to reduce cardiovascular risk. High SBP highlights the critical role of aggressive BP control, potentially through antihypertensive therapy and lifestyle modifications. Furthermore, modifiable factors such as smoking and increased waist circumference indicate the need for targeted behavioral interventions, including smoking cessation programs and weight management strategies. Based on the above findings, further work may be feasible for clinicians to stratify MAFLD patients into different risk categories by integrating these survival machine learning models and key predictors into the clinical early warning system and electronic health record (EHR) system. This measurement enables the implementation of personalized intervention strategies and enhances clinical decision support in real-world settings to reduce mortality risk in MAFLD patients, including intensified follow-up, medication therapy, and multidisciplinary care coordination. For instance, a risk prediction module could be embedded within the EHR system to automatically calculate mortality risk scores using routinely collected clinical and laboratory data, and identify individuals with MAFLD at high risk of mortality, prompting clinicians to consider earlier interventions, specialist referrals, or personalized care plans. Future implementation requires validation in diverse healthcare settings, and more importantly, needs consideration of ethical and regulatory aspects related to automated risk scoring. With further development, these tools could serve as a foundation for dynamic, data-driven risk assessment in MAFLD management.

Despite the contributions of our study, several limitations warrant consideration. First, this study included 1096 MAFLD patients sourced from the NHANES database for the years 2011 to 2014. However, data on C-reactive protein were unavailable for this period, and we treated the missing C-reactive protein data as absent and did not perform imputation. Meanwhile, within the 1096 individuals, only 2 (0.18%) required C-reactive protein as a necessary diagnostic criterion, suggesting that their exclusion is unlikely to introduce significant bias into the results of this study. Second, the absence of certain C-reactive protein data precluded its inclusion in our modeling and variable importance assessments. Nonetheless, existing literature indicates a potential relationship between C-reactive protein levels and the mortality rates associated with MAFLD^66^. Thus, further research is necessary to elucidate this connection in future investigations. Third, due to inherent limitations of data from the US-based NHANES cohort, our models lack external validation, which may compromise their generalizability. Although the NHANES is nationally representative, it features a specific demographic and ethnic composition and is situated within the context of the US healthcare system. Consequently, variations in genetic backgrounds, lifestyle, dietary habits, comorbidity prevalence, and healthcare access across global populations may affect the performance and calibration of our models. For instance, the predictive contribution of variables such as socioeconomic status or specific biomarkers may be different in populations with different healthcare environments or ethnic groups. Nonetheless, in our study, the SHAP value of ethnicity was only 0.0018 in the all-cause mortality model and 0.0022 in the CSD mortality model in the overall analyses, ranking thirteenth among all predictors (Fig. 4). In subgroup analyses, its importance fell beyond the top fifteenth (Fig. 5). These results suggest that ethnicity differences may have a limited impact on the broader applicability of our models. Despite this, external validation in diverse, independent cohorts from different geographical regions (e.g., Asia or Europe) and healthcare settings remains essential before these models can be confidently generalized. Future work should prioritize such validation efforts and explore potential region-specific adaptations to enhance model applicability across varied populations.

## Conclusions

In this study, we built five optimal mortality prediction models for patients with MAFLD, and all the models showed excellent predictive ability for predicting mortality. We also demonstrated that age, gender, smoking status, and other clinical variables were key factors in predicting survival during the follow-up in MAFLD patients. Due to limitations in the available data, we did not conduct external validation of the results. Future validation across various research centers will be essential for confirming the reliability of the model and enhancing the efficiency of the validation process.

## Supplementary Information

Below is the link to the electronic supplementary material.

Supplementary Material 1

## Data Availability

The datasets ANALYZED for this study can be found in the NHANES (https://www.cdc.gov/nchs/).
