# Supplementary material for: Survival machine learning models for predicting all-cause and case-specific mortality risk in metabolic dysfunction-associated fatty liver disease patients
Source: Sci Rep. 2025 Nov 28;15:42675. doi: 10.1038/s41598-025-26729-z (PMC12663551; doi:10.1038/s41598-025-26729-z)
Supplement: Supplementary file 1 — Supplementary Material 1 [file 41598_2025_26729_MOESM1_ESM.docx]

**Supplementary Table 1** Baseline characteristics for all-cause mortality among middle-aged participants

| **Variables** | **Total (n=1,962)** | **Alive (n=1,804)** | **Dead (n=158)** | ***P*** |
| --- | --- | --- | --- | --- |
| Gender (n, %) |  |  |  | 0.007 |
| Male | 821 (41.85%) | 771 (42.74%) | 50 (31.65%) |  |
| Female | 1,141 (58.15%) | 1,033 (57.26%) | 108 (68.35%) |  |
| Ethnicity (n, %) |  |  |  |  |
| Mexican American | 552 (28.13%) | 510 (25.27%) | 42 (26.58%) | 0.002 |
| Other Hispanic | 231 (11.77%) | 223 (12.36%) | 8 (5.06%) |  |
| Non-Hispanic White | 783 (39.91%) | 699 (38.75%) | 84 (53.16%) |  |
| Non-Hispanic Black | 221 (11.26%) | 205 (11.36%) | 16 (10.14%) |  |
| Other Race-Including Multi-Racial | 175 (8.93%) | 167 (9.27%) | 8 (5.06%) |  |
| Education level (n, %) |  |  |  | 0.049 |
| Below high school | 628 (32.01%) | 567 (31.43%) | 61 (38.61%) |  |
| High school | 395 (20.13%) | 359 (19.90%) | 36 (22.78%) |  |
| Above high school | 939 (47.86%) | 878 (48.67%) | 61 (38.61%) |  |
| Married status (n, %) |  |  |  | 0.027 |
| Married/ Living with Partner | 1,308 (66.67%) | 1,217 (67.46%) | 91 (57.60%) |  |
| Widowed/ Divorced/ Separated | 440 (22.43%) | 398 (22.06%) | 42 (26.58%) |  |
| Never married | 214 (10.90%) | 189 (10.48%) | 25 (15.82%) |  |
| Income level (n, %) |  |  |  | <0.001 |
| Low income | 623 (31.75%) | 544 (30.16%) | 79 (50.00%) |  |
| Middle income | 760 (38.74%) | 704 (39.02%) | 56 (35.44%) |  |
| High income | 579 (29.51%) | 556 (30.82%) | 23 (14.56%) |  |
| Smoke status (n, %) |  |  |  | <0.001 |
| Non-smoker | 949 (48.37%) | 909 (50.39%) | 40 (25.32%) |  |
| Former-smoker | 517 (26.35%) | 472 (26.16%) | 45 (28.48%) |  |
| Current-smoker | 496 (25.28%) | 423 (23.45%) | 73 (46.20%) |  |
| Alcohol assumption (n, %) |  |  |  | <0.001 |
| No | 1,781 (90.77%) | 1,650 (91.46%) | 131 (82.91%) |  |
| Yes | 181 (9.23%) | 154 (8.54%) | 27 (17.09%) |  |
| BMI (n, %) |  |  |  | <0.001 |
| <18.5 kg/m^2^ | 3 (0.15%) | 1 (0.06%) | 2 (1.27%) |  |
| 18.5-25 kg/m^2^ | 112 (5.71%) | 92 (5.10%) | 20 (12.66%) |  |
| 25-30 kg/m^2^ | 594 (30.28%) | 542 (30.04%) | 52 (32.91%) |  |
| ≥30 kg/m^2^ | 1,253 (63.86%) | 1,169 (64.80%) | 84 (53.16%) |  |
| Chronic diseases (n, %) |  |  |  |  |
| Hypertension | 849 (43.27%) | 761 (42.18%) | 88 (55.70%) | 0.001 |
| Congestive heart failure | 49 (2.50%) | 37 (2.05%) | 12 (7.59%) | <0.001 |
| Coronary heart disease | 55 (2.80%) | 45 (2.49%) | 10 (6.33%) | 0.005 |
| Angina | 44 (2.24%) | 35 (1.94%) | 9 (5.70%) | 0.002 |
| Heart attack | 67 (3.41%) | 56 (3.10%) | 11 (6.96%) | 0.010 |
| Stroke | 58 (2.96%) | 44 (2.44%) | 14 (8.86%) | <0.001 |
| Diabetes | 378 (19.27%) | 333 (18.46%) | 45 (28.48%) | 0.002 |
| Kidney disease | 65 (3.31%) | 54 (2.99%) | 11 (6.96%) | 0.008 |
| Asthma | 265 (13.51%) | 236 (13.08%) | 29 (18.35%) | 0.063 |
| Arthritis | 616 (31.40%) | 554 (30.71%) | 62 (39.24%) | 0.027 |
| Thyroid problem | 180 (9.17%) | 170 (9.42%) | 10 (6.33%) | 0.196 |
| Cancer | 118 (6.01%) | 100 (5.54%) | 18 (11.39%) | 0.003 |
| Hepatitis B (n, %) | 11 (0.56%) | 10 (0.55%) | 1 (0.63%) | 0.899 |
| Hepatitis C (n, %) | 89 (4.54%) | 68 (3.77%) | 21 (13.29%) | <0.001 |
| Physical activity (n, %) | 1,164 (59.33%) | 1,072 (59.42%) | 92 (58.23%) | 0.769 |
| Age (yrs) | 49.74±5.67 | 49.58±5.69 | 51.54±5.12 | <0.001 |
| Sleep (hours) | 7.03±1.39 | 7.05±1.39 | 6.87±1.38 | 0.120 |
| Waist circumference (cm) | 109.89±14.15 | 109.96±14.01 | 109.16±15.66 | 0.494 |
| SBP (mmHg) | 126.01±16.03 | 125.68±15.75 | 129.72±18.58 | 0.002 |
| DBP (mmHg) | 76.57±10.78 | 76.56±10.70 | 76.66±11.63 | 0.910 |
| Pulse (times per minute) | 73.57±12.34 | 73.47±12.25 | 74.78±13.29 | 0.198 |
| Platelet (1000 cells/uL) | 250.36±67.89 | 250.23±66.28 | 251.80±84.28 | 0.781 |
| ALT (U/L) | 34.23±31.32 | 35.00±31.46 | 37.85±29.67 | 0.273 |
| AST (U/L) | 30.34±31.83 | 29.95±32.03 | 34.78±29.18 | 0.068 |
| Total bilirubin (mg/dL) | 0.65±0.30 | 0.65±0.30 | 0.69±0.31 | 0.143 |
| GGT (U/L) | 54.41±84.86 | 51.59±80.04 | 86.59±123.49 | <0.001 |
| Albumin (g/L) | 41.94±3.35 | 41.97±3.29 | 41.66±4.02 | 0.269 |
| Triglyceride (mg/dL) | 182.16±142.23 | 179.40±141.10 | 213.61±151.56 | 0.004 |
| TC (mg/dL) | 204.78±43.76 | 204.89±43.35 | 203.49±48.33 | 0.700 |
| HDL-C (mg/dL) | 44.38±15.13 | 44.70±14.57 | 40.67±20.12 | 0.001 |
| LDL-C (mg/dL) | 122.88±37.00 | 123.41±36.69 | 116.89±40.05 | 0.034 |
| HbA1c (%) | 6.22±1.54 | 6.19±1.47 | 6.62±2.16 | 0.001 |
| Fasting Glu (mg/dL) | 125.73±51.20 | 124.13±47.75 | 143.90±78.72 | <0.001 |
| Fasting insulin (pmol/L) | 136.03±162.82 | 135.09±165.04 | 146.78±134.92 | 0.387 |
| Creatinine (mg/dL) | 0.84±0.36 | 0.82±0.22 | 1.00±1.01 | <0.001 |
| Uric acid (mg/dL) | 5.88±1.44 | 5.86±1.43 | 6.12±1.55 | 0.033 |
| BUN (mg/dL) | 13.26±5.07 | 13.18±4.71 | 14.19±±8.03 | 0.016 |

**Supplementary Table 2** Baseline characteristics for all-cause mortality among older participants

| **Variables** | **Total (n=2,453)** | **Alive (n=1,670)** | **Dead (n=783)** | ***P*** |
| --- | --- | --- | --- | --- |
| Gender (n, %) |  |  |  | <0.001 |
| Male | 1,086 (44.27%) | 803 (48.08%) | 283 (36.14%) |  |
| Female | 1,367 (55.73%) | 867 (51.92%) | 500 (63.86%) |  |
| Ethnicity (n, %) |  |  |  | <0.001 |
| Mexican American | 563 (22.95%) | 400 (23.95%) | 163 (20.82%) |  |
| Other Hispanic | 252 (10.27%) | 210 (12.57%) | 42 (5.36%) |  |
| Non-Hispanic White | 1,227 (50.03%) | 749 (44.85%) | 478 (61.05%) |  |
| Non-Hispanic Black | 263 (10.72%) | 184 (11.02%) | 79 (10.09%) |  |
| Other Race-Including Multi-Racial | 148 (6.03%) | 127 (7.61%) | 21 (2.68%) |  |
| Education level (n, %) |  |  |  | <0.001 |
| Below high school | 927 (37.79%) | 574 (34.37%) | 353 (45.08%) |  |
| High school | 551 (22.46%) | 366 (21.92%) | 185 (23.63%) |  |
| Above high school | 975 (39.75%) | 730 (43.71%) | 245 (31.29%) |  |
| Married status (n, %) |  |  |  | <0.001 |
| Married/ Living with Partner | 1,562 (63.68%) | 1,117 (66.89%) | 445 (56.83%) |  |
| Widowed/ Divorced/ Separated | 796 (32.45%) | 484 (28.98%) | 312 (39.85%) |  |
| Never married | 95 (3.87%) | 69 (4.13%) | 26 (3.32%) |  |
| Income level (n, %) |  |  |  | <0.001 |
| Low income | 710 (28.94%) | 463 (27.72%) | 247 (31.55%) |  |
| Middle income | 1,108 (45.17%) | 715 (42.82%) | 393 (50.19%) |  |
| High income | 635 (25.89%) | 492 (29.46%) | 143 (18.26%) |  |
| Smoke status (n, %) |  |  |  | <0.001 |
| Non-smoker | 1,121 (45.70%) | 811 (48.56%) | 310 (39.59%) |  |
| Former-smoker | 1,079 (43.99%) | 698 (41.80%) | 381 (48.66%) |  |
| Current-smoker | 253 (10.31%) | 161 (9.64%) | 92 (11.75%) |  |
| Alcohol assumption (n, %) |  |  |  | 0.058 |
| No | 2,304 (93.93%) | 1,579 (94.55%) | 725 (92.59%) |  |
| Yes | 149 (6.07%) | 91 (5.45%) | 58 (7.41%) |  |
| BMI (n, %) |  |  |  | <0.001 |
| <18.5 kg/m^2^ | 2 (0.08%) | 0 (0.00%) | 2 (0.26%) |  |
| 18.5-25 kg/m^2^ | 180 (7.34%) | 97 (5.81%) | 83 (10.60%) |  |
| 25-30 kg/m^2^ | 889 (36.24%) | 565 (33.83%) | 324 (41.38%) |  |
| ≥30 kg/m^2^ | 1,382 (56.34%) | 1,008 (60.36%) | 374 (47.76%) |  |
| Chronic diseases (n, %) |  |  |  |  |
| Hypertension | 1,646 (67.10%) | 1,123 (67.25%) | 523 (66.79%) | 0.825 |
| Congestive heart failure | 220 (8.97%) | 110 (6.59%) | 110 (14.05%) | <0.001 |
| Coronary heart disease | 299 (12.19%) | 177 (10.60%) | 122 (15.58%) | <0.001 |
| Angina | 185 (7.54%) | 94 (5.63%) | 91 (11.62%) | <0.001 |
| Heart attack | 282 (11.50%) | 154 (9.22%) | 128 (16.35%) | <0.001 |
| Stroke | 182 (7.42%) | 93 (5.57%) | 89 (11.37%) | <0.001 |
| Diabetes | 773 (31.51%) | 536 (32.10%) | 237 (30.27%) | 0.364 |
| Kidney disease | 139 (5.67%) | 89 (5.33%) | 50 (6.39%) | 0.291 |
| Asthma | 323 (13.17%) | 231 (13.83%) | 92 (11.75%) | 0.155 |
| Arthritis | 1,244 (50.71%) | 821 (49.16%) | 423 (54.02%) | 0.025 |
| Thyroid problem | 391 (15.94%) | 295 (17.66%) | 96 (12.26%) | 0.001 |
| Cancer | 464 (18.92%) | 292 (17.49%) | 172 (21.97%) | 0.008 |
| Hepatitis B (n, %) | 10 (0.41%) | 7 (0.42%) | 3 (0.38%) | 0.896 |
| Hepatitis C (n, %) | 37 (1.51%) | 28 (1.68%) | 9 (1.15%) | 0.318 |
| Physical activity (n, %) | 1,163 (47.41%) | 813 (48.68%) | 350 (44.70%) | 0.066 |
| Age (yrs) | 69.81±6.78 | 68.20±6.13 | 73.24±6.82 | <0.001 |
| Sleep (hours) | 7.38±1.47 | 7.40±1.51 | 7.34±1.37 | 0.384 |
| Waist circumference (cm) | 109.48±12.45 | 109.96±12.42 | 108.45±12.46 | 0.005 |
| SBP (mmHg) | 134.33±19.54 | 133.02±18.19 | 137.14±21.81 | <0.001 |
| DBP (mmHg) | 68.27±12.43 | 69.30±11.84 | 66.08±13.34 | <0.001 |
| Pulse (times per minute) | 70.55±11.88 | 69.86±11.06 | 72.03±13.35 | <0.001 |
| Platelet (1000 cells/uL) | 231.30±64.28 | 232.10±65.53 | 229.60±65.86 | 0.368 |
| ALT (U/L) | 26.31±17.73 | 26.79±16.41 | 25.29±20.23 | 0.051 |
| AST (U/L) | 26.52±13.65 | 26.00±11.88 | 27.65±16.77 | 0.005 |
| Total bilirubin (mg/dL) | 0.70±0.30 | 0.67±0.28 | 0.75±0.32 | <0.001 |
| GGT (U/L) | 39.72±43.77 | 38.21±42.81 | 42.94±45.61 | 0.013 |
| Albumin (g/L) | 41.67±3.24 | 41.65±3.19 | 41.72±3.35 | 0.631 |
| Triglyceride (mg/dL) | 160.47±91.58 | 157.18±88.67 | 167.49±97.17 | 0.009 |
| TC (mg/dL) | 190.73±43.04 | 189.44±42.51 | 193.46±44.04 | 0.031 |
| HDL-C (mg/dL) | 45.20±17.64 | 47.12±15.01 | 41.12±21.68 | <0.001 |
| LDL-C (mg/dL) | 109.80±36.84 | 109.22±36.58 | 111.02±37.38 | 0.260 |
| HbA1c (%) | 6.35±1.32 | 6.36±1.28 | 6.33±1.40 | 0.700 |
| Fasting Glu (mg/dL) | 130.57±46.08 | 129.83±44.46 | 132.14±49.34 | 0.246 |
| Fasting insulin (pmol/L) | 135.16±154.51 | 136.75±163.00 | 131.78±134.67 | 0.458 |
| Creatinine (mg/dL) | 0.97±0.44 | 0.94±0.40 | 1.04±0.51 | <0.001 |
| Uric acid (mg/dL) | 6.10±1.49 | 6.02±1.42 | 6.27±1.62 | <0.001 |
| BUN (mg/dL) | 16.84±6.96 | 16.13±6.16 | 18.36±8.22 | <0.001 |

**Supplementary Table 3** Baseline characteristics for CSD mortality among older participants

| **Variables** | **Total (n=2,453)** | **Alive (n=2,196)** | **Dead (n=257)** | ***P*** |
| --- | --- | --- | --- | --- |
| Gender (n, %) |  |  |  | 0.009 |
| Male | 1,086 (44.27%) | 992 (45.17%) | 94 (36.58%) |  |
| Female | 1,367 (55.73%) | 1,204 (54.83%) | 163 (63.42%) |  |
| Ethnicity (n, %) |  |  |  | 0.001 |
| Mexican American | 563 (22.95%) | 511 (23.27%) | 52 (20.23%) |  |
| Other Hispanic | 252 (10.27%) | 238 (10.84%) | 14 (5.45%) |  |
| Non-Hispanic White | 1,227 (50.03%) | 1,071 (48.77%) | 156 (60.70%) |  |
| Non-Hispanic Black | 263 (10.72%) | 235 (10.70%) | 28 (10.89%) |  |
| Other Race-Including Multi-Racial | 148 (6.03%) | 141 (6.42%) | 7 (2.72%) |  |
| Education level (n, %) |  |  |  | 0.008 |
| Below high school | 927 (37.79%) | 811 (36.93%) | 116 (45.14%) |  |
| High school | 551 (22.46%) | 490 (22.31%) | 61 (23.74%) |  |
| Above high school | 975 (39.75%) | 895 (40.76%) | 80 (31.13%) |  |
| Married status (n, %) |  |  |  | 0.003 |
| Married/ Living with Partner | 1,562 (63.68%) | 1,423 (64.80%) | 139 (54.09%) |  |
| Widowed/ Divorced/ Separated | 796 (32.45%) | 690 (31.42%) | 106 (41.24%) |  |
| Never married | 95 (3.87%) | 83 (3.78%) | 12 (4.67%) |  |
| Income level (n, %) |  |  |  | 0.006 |
| Low income | 710 (28.94%) | 636 (28.96%) | 74 (28.79%) |  |
| Middle income | 1,108 (45.17%) | 972 (44.26%) | 136 (52.92%) |  |
| High income | 635 (25.89%) | 588 (26.78%) | 47 (18.29%) |  |
| Smoke status (n, %) |  |  |  | 0.652 |
| Non-smoker | 1,121 (45.70%) | 1,009 (45.95%) | 112 (43.58%) |  |
| Former-smoker | 1,079 (43.99%) | 959 (43.67%) | 120 (46.69%) |  |
| Current-smoker | 253 (10.31%) | 228 (10.38%) | 25 (9.73%) |  |
| Alcohol assumption (n, %) |  |  |  | 0.226 |
| No | 2,304 (93.93%) | 2,067 (94.13%) | 237 (92.22%) |  |
| Yes | 149 (6.07%) | 129 (5.87%) | 20 (7.78%) |  |
| BMI (n, %) |  |  |  | 0.280 |
| <18.5 kg/m^2^ | 2 (0.08%) | 1 (0.05%) | 1 (0.39%) |  |
| 18.5-25 kg/m^2^ | 180 (7.34%) | 162 (7.38%) | 18 (7.00%) |  |
| 25-30 kg/m^2^ | 889 (36.24%) | 791 (36.01%) | 98 (38.13%) |  |
| ≥30 kg/m^2^ | 1,382 (56.34%) | 1,242 (56.56%) | 140 (54.48%) |  |
| Chronic diseases (n, %) |  |  |  |  |
| Hypertension | 1,646 (67.10%) | 1,460 (66.48%) | 186 (72.37%) | 0.057 |
| Congestive heart failure | 220 (8.97%) | 189 (8.61%) | 31 (12.06%) | 0.067 |
| Coronary heart disease | 299 (12.19%) | 254 (11.57%) | 45 (17.51%) | 0.006 |
| Angina | 185 (7.54%) | 152 (6.92%) | 33 (12.84%) | 0.001 |
| Heart attack | 282 (11.50%) | 242 (11.02%) | 40 (15.56%) | 0.031 |
| Stroke | 182 (7.42%) | 151 (6.88%) | 31 (12.06%) | 0.003 |
| Diabetes | 773 (31.51%) | 686 (31.24%) | 87 (33.85%) | 0.393 |
| Kidney disease | 139 (5.67%) | 119 (5.42%) | 20 (7.78%) | 0.121 |
| Asthma | 323 (13.17%) | 289 (13.16%) | 34 (13.23%) | 0.975 |
| Arthritis | 1,244 (50.71%) | 1,107 (50.41%) | 137 (53.31%) | 0.379 |
| Thyroid problem | 391 (15.94%) | 360 (16.39%) | 31 (12.06%) | 0.073 |
| Cancer | 464 (18.92%) | 419 (19.08%) | 45 (17.51%) | 0.543 |
| Hepatitis B (n, %) | 10 (0.41%) | 10 (0.46%) | 0 (0.00%) | 0.278 |
| Hepatitis C (n, %) | 37 (1.51%) | 35 (1.59%) | 2 (0.78%) | 0.310 |
| Physical activity (n, %) | 1,163 (47.41%) | 1,047 (47.68%) | 116 (45.14%) | 0.440 |
| Age (yrs) | 69.81±6.78 | 69.30±6.58 | 74.14±6.94 | <0.001 |
| Sleep (hours) | 7.38±1.47 | 7.37±1.48 | 7.49±1.33 | 0.224 |
| Waist circumference (cm) | 109.48±12.45 | 109.50±12.48 | 109.27±12.18 | 0.782 |
| SBP (mmHg) | 134.33±19.54 | 133.72±19.08 | 139.60±22.48 | <0.001 |
| DBP (mmHg) | 68.27±12.43 | 68.73±12.10 | 64.36±14.40 | <0.001 |
| Pulse (times per minute) | 70.55±11.88 | 70.48±11.80 | 71.14±12.56 | 0.400 |
| Platelet (1000 cells/uL) | 231.30±64.28 | 231.50±64.17 | 229.61±65.31 | 0.655 |
| ALT (U/L) | 26.31±17.73 | 26.37±16.46 | 25.79±26.24 | 0.619 |
| AST (U/L) | 26.52±13.65 | 26.41±12.46 | 27.47±21.23 | 0.240 |
| Total bilirubin (mg/dL) | 0.70±0.30 | 0.69±0.30 | 0.72±0.29 | 0.119 |
| GGT (U/L) | 39.72±43.77 | 39.78±44.56 | 39.16±36.36 | 0.830 |
| Albumin (g/L) | 41.67±3.24 | 41.65±3.23 | 41.82±3.31 | 0.432 |
| Triglyceride (mg/dL) | 160.47±91.58 | 159.11±90.29 | 172.04±101.33 | 0.032 |
| TC (mg/dL) | 190.73±43.04 | 190.38±42.98 | 193.65±43.49 | 0.249 |
| HDL-C (mg/dL) | 45.20±17.64 | 45.67±17.08 | 41.20±21.44 | <0.001 |
| LDL-C (mg/dL) | 109.80±36.84 | 109.74±36.88 | 110.25±36.57 | 0.834 |
| HbA1c (%) | 6.35±1.32 | 6.33±1.28 | 6.53±1.59 | 0.020 |
| Fasting Glu (mg/dL) | 130.57±46.08 | 129.71±44.85 | 137.87±55.05 | 0.007 |
| Fasting insulin (pmol/L) | 135.16±154.51 | 135.46±159.24 | 132.61±106.02 | 0.779 |
| Creatinine (mg/dL) | 0.97±0.44 | 0.96±0.44 | 1.04±0.48 | 0.004 |
| Uric acid (mg/dL) | 6.10±1.49 | 6.06±1.47 | 6.39±1.66 | 0.001 |
| BUN (mg/dL) | 16.84±6.96 | 16.60±6.74 | 18.91±8.36 | <0.001 |

**Supplementary Table 4 Performance of the models based on age for predicting all-cause and CSD mortality in overall analyses**

| **Group** | **Index** | **CoxPH** | **RSF** | **CoxNet** | **EST** | **GBS** |
| --- | --- | --- | --- | --- | --- | --- |
| **All-cause mortality** | **tAUC** | **0.795** | **0.771** | **0.795** | **0.771** | **0.789** |
|  | **C-index** | **0.753** | **0.740** | **0.753** | **0.740** | **0.750** |
|  | **IBS** | **0.115** | **0.121** | **0.115** | **0.121** | **0.116** |
| **CSD mortality** | **tAUC** | **0.816** | **0.762** | **0.816** | **0.768** | **0.805** |
|  | **C-index** | **0.774** | **0.741** | **0.774** | **0.743** | **0.766** |
|  | **IBS** | **0.061** | **0.063** | **0.061** | **0.062** | **0.061** |

**Supplementary Table 5 Performance of the models based on age for predicting all-cause and CSD mortality in subgroup analyses**

| **Group** | **Index** | **CoxPH** | **RSF** | **CoxNet** | **EST** | **GBS** |
| --- | --- | --- | --- | --- | --- | --- |
| **All-cause mortality for**  **middle-aged adults** | **tAUC** | **0.639** | **0.571** | **0.639** | **0.575** | **0.607** |
|  | **C-index** | **0.624** | **0.568** | **0.624** | **0.568** | **0.616** |
|  | **IBS** | **0.080** | **0.083** | **0.080** | **0.083** | **0.081** |
| **All-cause mortality for**  **older adults** | **tAUC** | **0.736** | **0.708** | **0.736** | **0.712** | **0.723** |
|  | **C-index** | **0.708** | **0.703** | **0.708** | **0.704** | **0.701** |
|  | **IBS** | **0.155** | **0.162** | **0.156** | **0.161** | **0.159** |
| **CSD mortality for**  **older adults** | **tAUC** | **0.791** | **0.723** | **0.791** | **0.732** | **0.769** |
|  | **C-index** | **0.743** | **0.715** | **0.743** | **0.713** | **0.737** |
|  | **IBS** | **0.093** | **0.102** | **0.093** | **0.100** | **0.097** |


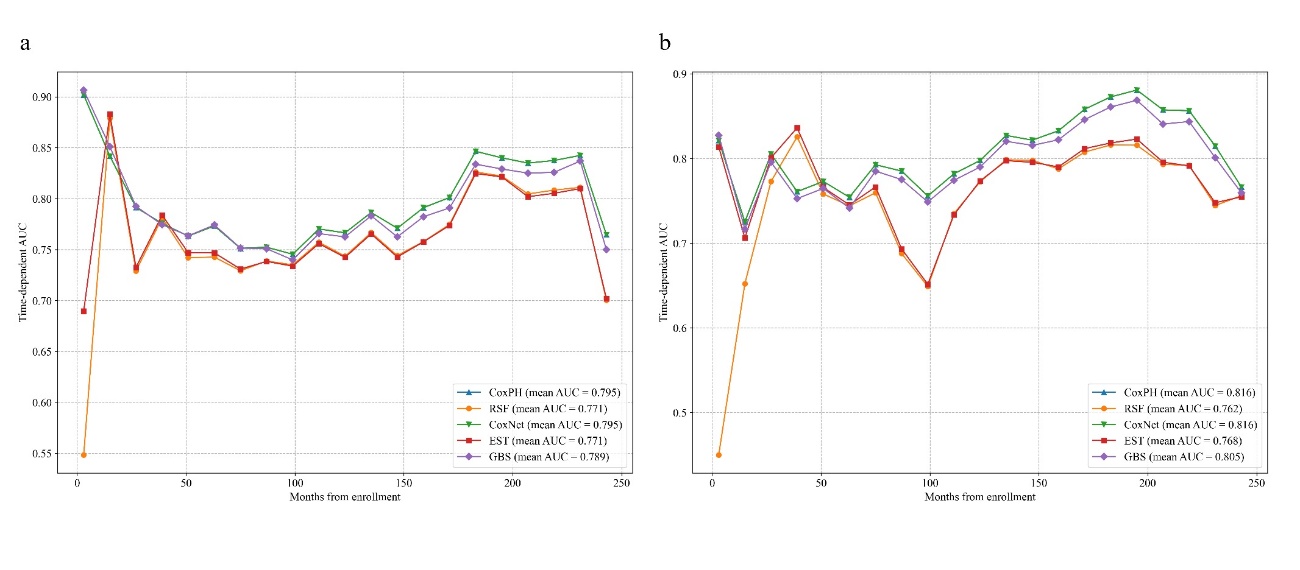


**Supplementary Figure 1 Time-dependent AUC of different models based on age for predicting mortality in the overall analyses. (a) models with all-cause mortality, (b) models with CSD mortality.**


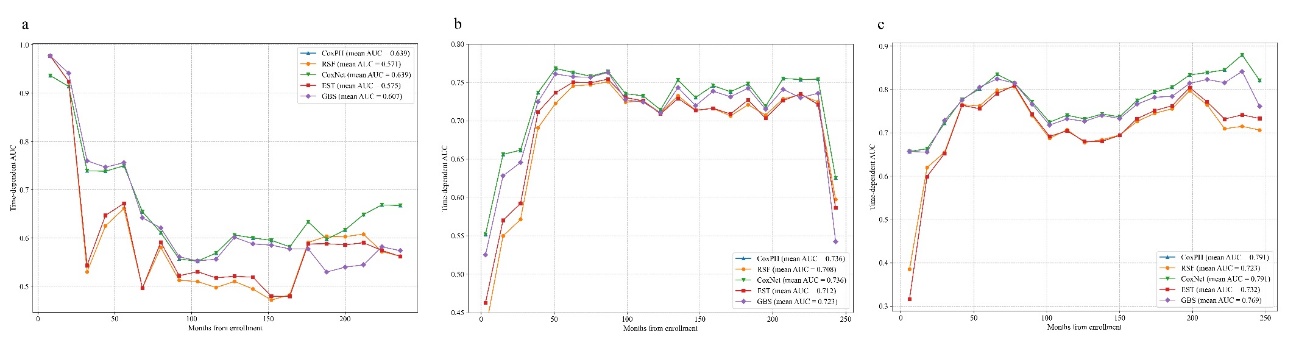


**Supplementary Figure 2 Time-dependent AUC of different models based on age for predicting mortality in the subgroup analyses. (a) models with all-cause mortality for middle-aged adults, (b) models with all-cause mortality for older adults, (c) models with CSD mortality for older adults.**
